# Saponin from Tea (*Camellia sinensis*) Seed Meal Attenuates Cortisol-Induced Lipogenesis and Inflammation in Human Cells

**DOI:** 10.3390/molecules30193844

**Published:** 2025-09-23

**Authors:** Jian Li, Lu-Yao Zhang, Yuan-Cheng Huang, Jian-Ming Deng, Min Yu, Christos C Zouboulis, Jin-Hua Li, Guang-Li Wang, Jing Wang

**Affiliations:** 1 School of Chemical and Material Engineering, Jiangnan University, Wuxi 214122, China; 2 Guangzhou Huashi Cosmetic Technology Co., Ltd., Guangzhou 510000, China; 3 Department of Dermatology, Venereology, Allergology and Immunology, Staedtisches Klinikum Dessau, Brandenburg Medical School Theodor Fontane and Faculty of Health Sciences Brandenburg, D-06847 Dessau, Germany

**Keywords:** tea seed saponins, cortisol, 11β-HSD1, sebum control, anti-inflammatory

## Abstract

A fast-paced lifestyle contributes to heightened emotional stress, driving the demand for milder and safer cosmetic ingredients that can counteract stress-induced skin damage—a focus of cutting-edge research in the field. Aim: The aim was to elucidate the role and mechanistic basis of tea (*Camellia sinensis*) seed meal saponin (Sap) in regulating stress-induced sebum overproduction and inflammatory responses. Methods: The composition and chemical structure of Sap were analyzed using UV-vis absorption spectroscopy, Fourier-transform infrared spectroscopy (FT-IR), and ultra-high-performance liquid chromatography–mass spectrometry (UHPLC-MS). In vitro models of cortisone-induced excessive lipid accumulation and the tumor necrosis factor-alpha (TNF-α)-stimulated inflammatory models were established on sebaceous gland cells (SZ95) and normal human epidermal keratinocytes (NHEKs), respectively. Cortisol and inflammatory cytokine secretion levels in cells were detected using ELISA. Additionally, the signaling pathways were revealed by Western blot (WB) and real-time quantitative polymerase chain reaction (RT-PCR). Results: Five saponins were identified in the Sap extract, all belonging to the oleanolic-acid-type pentacyclic triterpenes. Sap treatment significantly attenuated cortisone-induced cortisol secretion and lipid accumulation in SZ95 sebocytes. Mechanistically, Sap inhibited the 11β-HSD1/SREBP-1 pathway, which mediates its sebosuppressive effects, while concurrently down-regulating the mRNA expression of key downstream transcription factors and enzymes, including SREBP-1, FAS, and ACC. Additionally, Sap treatment significantly attenuated TNF-α-stimulated cortisol secretion and inflammatory cytokine (IL-1β, IL-6, and IL-8) production in NHEK cells through the inhibition of the 11β-HSD1/TLR2/NF-κB signaling pathway. Conclusion: Sap demonstrated dual inhibitory effects, suppressing both emotional-stress-induced sebum overproduction and inflammatory cytokines secretion.

## 1. Introduction

In modern society, emotional stress has emerged as a prevalent concern that adversely affects both psychological well-being and cutaneous health. The hypothalamic–pituitary–adrenal (HPA) axis, a component of the central nervous system, serves as the primary regulator of stress responses [1]. Upon stress activation, the hypothalamus releases corticotropin-releasing hormone (CRH), which stimulates the pituitary secretion of adrenocorticotropic hormone (ACTH). This systemic cascade ultimately triggers glucocorticoid synthesis in the adrenal cortex [2,3]. Notably, cutaneous cells—including keratinocytes, fibroblasts, and sebocytes—can locally produce cortisol in response to CRH/ACTH signaling, profoundly influencing skin homeostasis [4,5]. Emerging evidence indicates that CRH production by sebaceous glands contributes to localized stress responses, exacerbating inflammatory skin conditions such as acne, rosacea, and premature aging [6,7,8,9,10,11]. 11β-hydroxysteroid dehydrogenase type 1 (11β-HSD1) regulates the conversion of inactive cortisone to active cortisol, thereby amplifying local cortisol levels during stress [12,13]. Chronic HPA axis activation and subsequent cortisol overproduction have been mechanistically linked to various dermatological pathologies, including skin barrier dysfunction [14], acne [15], hyperpigmentation [16], skin aging [17], and inflammation [4]. Despite these established connections, the development of 11β-HSD1 inhibitors as therapeutic cosmetic agents for stress-related dermatoses remains largely unexplored. The targeted modulation of this pathway may represent a promising strategy for maintaining skin homeostasis against psychogenic stressors.

Plant-derived extracts are gaining prominence in cosmetic formulations owing to their natural origin, favorable biocompatibility, and mild dermatological profiles. Growing evidence highlights their therapeutic potential in addressing stress-related skin conditions. For instance, iris pallor extract counteracts cortisol-induced reductions in type I collagen and hyaluronic acid synthesis in HaCaT cells [18]. Tephrosia purpurea extract attenuates cortisone-induced stress responses through cortisol inhibition [19]. Moreover, dark tea polysaccharides inhibit cortisone-induced cortisol secretion and reduce lipid production in SZ95 sebocytes, though their anti-lipogenic mechanism remains unclear [20]. These findings collectively hint at the prospect of plant exacts with potentially wide applications in stress-targeted skincare interventions.

While saponin (Sap) from tea (*Camellia sinensis*) seeds have established antioxidant, anti-inflammatory, and sebum-control effects [21,22,23,24], no report exists on their efficacy in regulating emotional-stress-induced cortisol overproduction. In this study, Sap from tea (Anhua, Hunan Province) seed meal was revealed, for the first time, to reduce cortisol secretion in human immortalized sebaceous gland cells (SZ95) and normal human epidermal keratinocytes (NHEKs) through the inhibition of the activity of 11β-HSD1. Moreover, the sebum suppression and anti-inflammatory signaling pathways caused by cortisol overproduction were investigated. Considering that emotional-stress-induced excessive sebum production and inflammatory reactions can lead to a deterioration in skin homeostasis, this study validates a new application of Sap to relieve emotional and local stress-induced detrimental skin problems.

## 2. Results

### 2.1. Characterization of Sap in Tea Seed Meal

**Figure 1:**
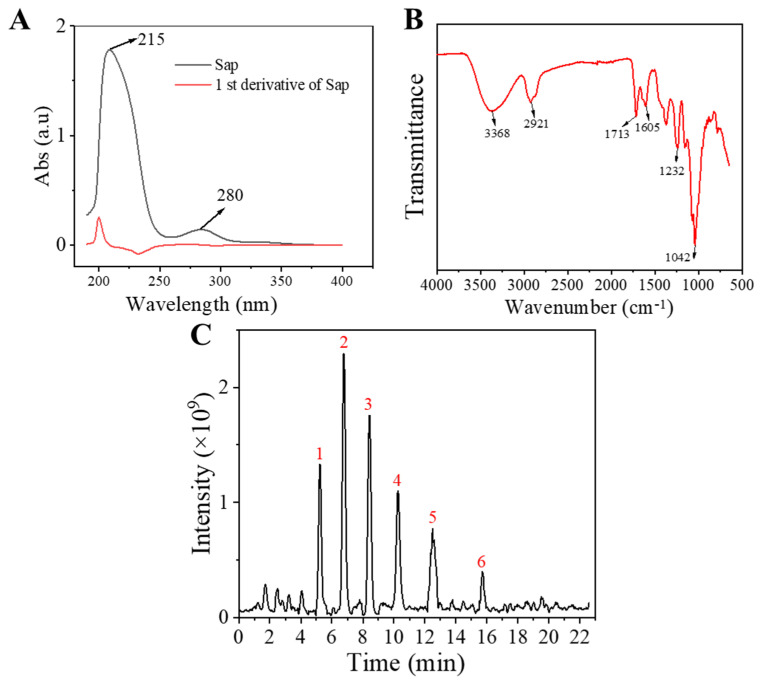
Structural characterization of Sap from tea seed meal: (**A**) UV-vis absorption spectrum and its corresponding first-order derivative curve; (**B**) FT-IR spectra of the obtained Sap from tea seed meal; (**C**) total ion flow diagram of Sap from tea seed meal in UHPLC.

The structural properties of the Sap were characterized through multi-spectroscopic approaches including UV-vis absorption spectroscopy, Fourier-transform infrared (FT-IR) spectroscopy, and ultra-high-performance liquid chromatography–mass spectrometry (UHPLC-MS). In the UV-vis spectrum, a prominent absorption peak at 215 nm was observed (Figure 1A), corresponding to the typical chromophore of triterpenoid saponins [25,26]. A minor peak at 280 nm likely originated from trace amounts of cinnamic acid or flavonoid residues [27,28]. To refine the location of the maximal absorption and to evaluate spectral symmetry, the first-derivative trace was calculated (Figure 1A). Its zero-crossing coincided exactly with 215 nm, and the narrow peak-to-trough width (≈6 nm), without additional zero-crossings between 220 nm and 245 nm, confirmed a single major chromophore, corroborating that the dominant constituent was a triterpenoid saponin. Moreover, the contents of saponins and polyphenols in the Sap were determined by the vanillin-concentrated sulfuric acid method and the Folin–Ciocalteu assay, which showed that the contents of saponins and polyphenols were 94.83% and 1.23%, respectively. 

The FT-IR spectral profile of the isolated Sap (Figure 1B) exhibited characteristic absorption bands that were in good agreement with literature reports for tea seed saponins [25,27,28]. Key vibrational modes were identified as follows: characteristic peaks at 3371 and 2923 cm^−1^ were attributed to -OH and C-H stretching vibrations, respectively. Peaks at 1715 and 1609 cm^−1^ corresponded to C=O stretching vibrations, while those at 1234 and 1038 cm^−1^ were assigned to C-O-H bending and C-O-C stretching vibrations, respectively.

The molecular composition of the obtained Sap was further elucidated using UPLC-MS in positive ionization mode. The total ion chromatogram (TIC) of the Sap exhibited five predominant peaks (Figure 1C), with their corresponding mass spectral data systematically analyzed and presented in Table 1. Fragment ions were compared against the MMSs-public-Expbioinsilico-pos-VS17 and MMSs-pos-Vanya-Fiehn Natural Products Library databases, and the PubChem database (https://pubchem.ncbi.nlm.nih.gov/, accessed on 1 September 2025. Our analysis identified five distinct saponin compounds (Table 1), all belonging to the oleanane-type pentacyclic triterpenoid family with varying degrees of glycosylation and different aglycone types [29,30,31]. For specific information on molecular mass spectrometry fragments, please refer to the Supplementary Materials cited.

### 2.2. Sap Inhibits Cortisone-Induced Cortisol Secretion and Lipid Production in SZ95 Sebocytes

#### 2.2.1. Effects of Sap, Cortisone, and Metyrapone on SZ95 Sebocyte Viability

**Figure 2:**
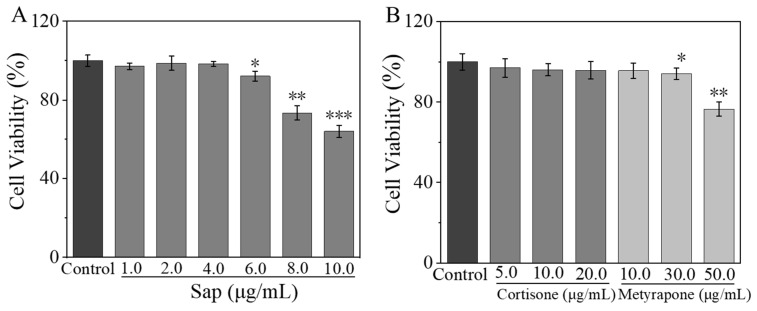
Effects of Sap (**A**), cortisone, and metyrapone (**B**) on cell viability of SZ95 sebocytes. The error line represents the standard deviation of three independent culture results (*, ** and *** indicate *p* < 0.05, 0.01 and 0.001 vs. control group).

An MTT assay was performed to determine the cytotoxicity of Sap, cortisone, and metyrapone on SZ95 sebocytes. The cell viability remained above 90% when the concentration range of Sap was within 1.0–6.0 μg/mL; however, it dropped below 80% at concentrations of 8.0 and 10.0 μg/mL (Figure 2A). Furthermore, cortisone maintained a cell viability higher than 90% within the concentration ranges of 5.0–20.0 μg/mL. While metyrapone also kept the cell viability above 90% in the concentration ranges of 10.0–30.0 μg/mL, the activity decreased below 80% at a high concentration of 50.0 μg/mL (Figure 2B). Therefore, the concentrations of ≤ 6.0 μg/mL for Sap, ≤ 20.0 μg/mL for cortisone, and ≤ 30.0 μg/mL for metyrapone were selected for subsequent experiments.

#### 2.2.2. Inhibitive Effect of Sap on Cortisone-Induced Cortisol and Lipid Secretion

**Figure 3:**
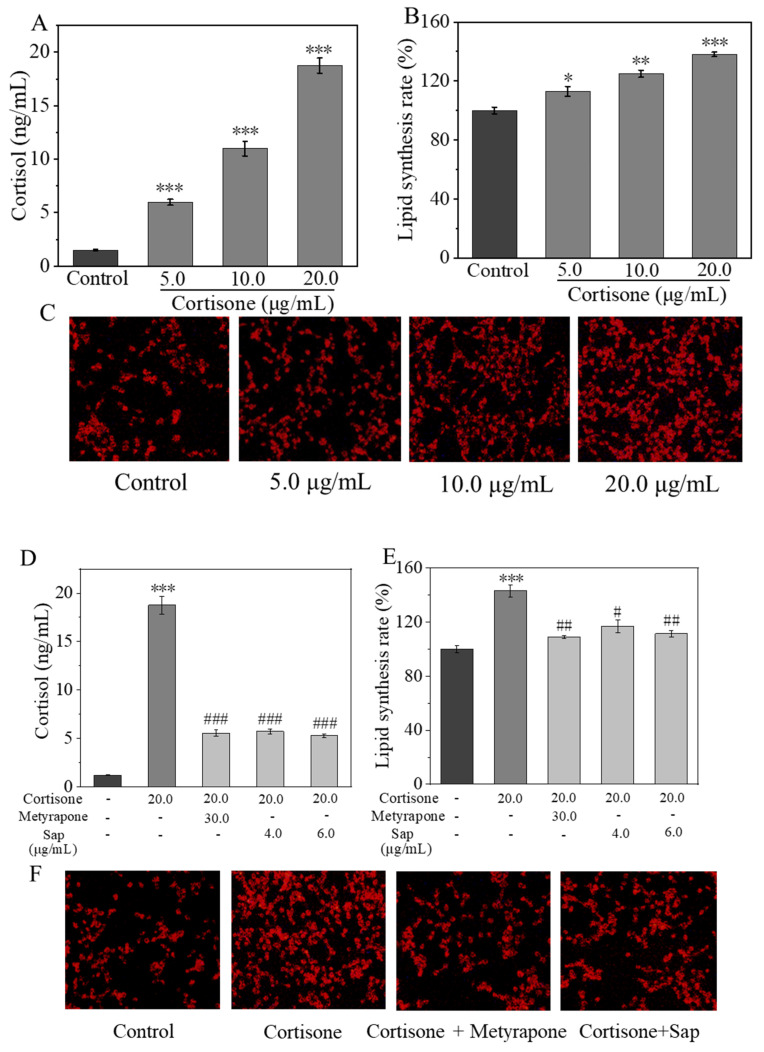
Cortisone promotes cortisol (**A**) and lipid production (**B**,**C**) in SZ95 sebocytes, while Sap inhibits cortisone-induced cortisol secretion (**D**) and lipid production (**E**,**F**). (**B**) presents the quantitative analysis of fluorescence intensity corresponding to (**C**). Among them, (**B**) presents the quantitative analysis of fluorescence intensity corresponding to (**C**). The error line represents the standard deviation of three independent culture results (*, ** and *** indicate *p* < 0.05, 0.01 and 0.001 vs. control group; #, ##, and ### indicate *p* < 0.05, 0.01, and 0.001 vs. cortisone group).

To simulate a stress environment, SZ95 sebocytes were treated with cortisone, and the levels of cortisol in the cell culture supernatant as well as intracellular lipid production were measured. As shown in Figure 3A, cortisone increased cortisol production in SZ95 cells in a concentration-dependent manner, with a cortisol concentration of 18.7 ng/mL in the cell supernatant at a cortisone concentration of 20.0 μg/mL. Moreover, the lipid content within the cells determined using Nile Red staining (as depicted in Figure 3B,C) also revealed that cortisone increased the lipid production in SZ95 sebocytes in a concentration-dependent manner. Therefore, we can see that cortisone can promote the production of both cortisol and lipids in SZ95 cells. Subsequent experiments utilized a cortisone concentration of 20.0 μg/mL as a stimulant to investigate whether Sap can inhibit cortisone-induced cortisol and lipid production.

The 11β-HSD1 enzyme converts inactive cortisone to the active form of cortisol, which, in turn, regulates glucocorticoid-mediated lipid synthesis and plays an important role in steroid-induced and stress-related acne [32]. Metyrapone is a known drug that inhibits the synthesis of corticosteroids, and its main effect is to reduce the conversion of inactive cortisone to active cortisol by inhibiting 11β-HSD1 enzyme activity [33]. In our experiment, SZ95 sebocytes were co-cultured with the mixture of cortisone and Sap or metyrapone (as positive control), respectively, to investigate the effects of Sap on cortisone-induced cortisol and lipid production by adopting the ELISA and Nile Red staining as evaluation methods. 

The ELISA results showed that Sap significantly inhibited cortisone-induced cortisol conversion, and its inhibitory effect was comparable to that of the positive metyrapone group (Figure 3D). The results of Nile Red staining are shown in Figure 3E,F. Compared with the control group, the fluorescence intensity of the cortisone group was significantly enhanced, with the lipid synthesis rate increased by 43%. On the contrary, the fluorescence intensity of the Sap group (6.0 μg/mL, incubation time: 24 h, cell viability > 90%) was significantly reduced, and the lipid synthesis rate of the Sap group was 32% lower than that of the cortisone stimulative group. Meanwhile, the inhibitory effect of Sap was comparable to that of the metyrapone group.

### 2.3. Sap Modulates Lipogenesis in SZ95 Sebocytes via the 11β-HSD1/SREBP-1 Pathways

**Figure 4:**
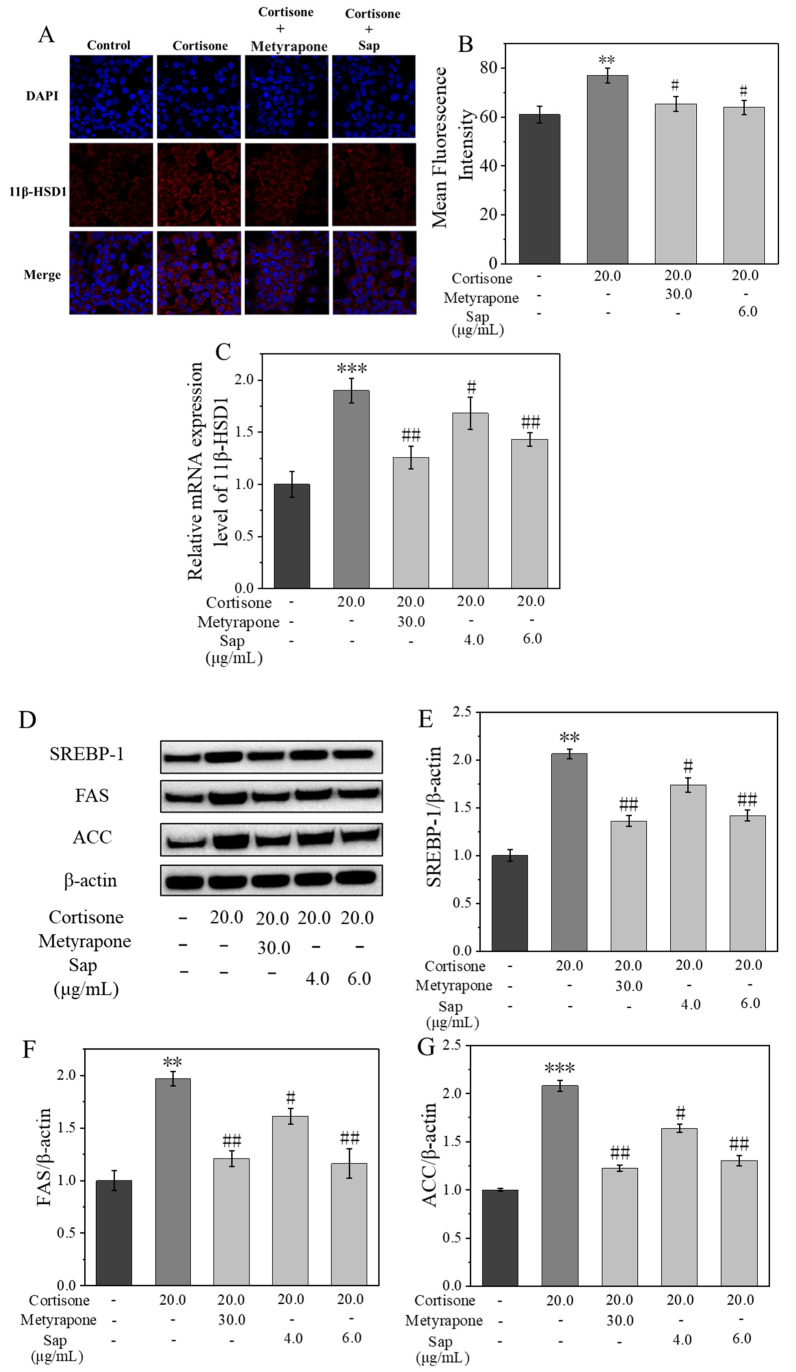

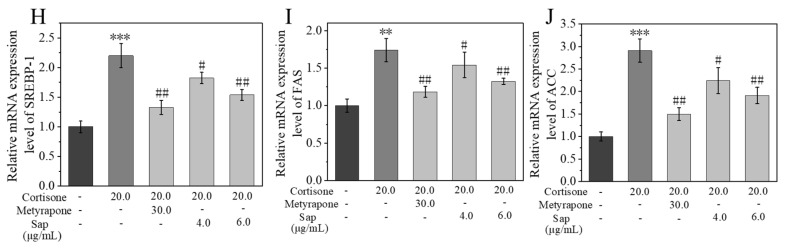
Sap inhibits cortisone-induced 11β-HSD1 enzyme expression. (**A**,**B**) 11β-HSD1 enzyme protein expression measured by immunostaining; (**B**) presents the quantitative analysis of mean fluorescence intensity corresponding to (**A**). (**C**) 11β-HSD1 mRNA expression measured by RT-PCR. Sap inhibits the protein and mRNA expressions of SREBP-1, FAS, and ACC in cortisone-stimulated SZ95 sebocytes. (**D**) Western blot protein bands; (**E**–**G**) relative protein expression levels; (**H**–**J**) relative mRNA expression levels. The error line represents the standard deviation of three independent culture results (** and *** indicate *p* < 0.01 and 0.001 vs. control group; # and ## indicate *p* < 0.05 and 0.01 vs. cortisone group).

The expression of 11β-HSD1 in SZ95 sebocytes [34] was evaluated by immunofluorescence staining and RT-PCR (shown in Figure 4A,C). Compared with the cortisone group, 11β-HSD1 enzyme expression in the Sap group and metyrapone group was significantly decreased. Specifically, the expression level of 11β-HSD1 mRNA in the Sap group (6.0 μg/mL, incubation time: 24 h, cell viability > 90%) was reduced by 47% compared with the cortisone group, indicating that Sap effectively reduced cortisol production by inhibiting 11β-HSD1 expression in SZ95 sebocytes.

The results demonstrated that Sap inhibited the expression of the 11β-HSD1 enzyme, thereby effectively reducing the conversion of cortisone to cortisol. However, the specific mechanism by which Sap inhibited lipogenesis remained to be fully elucidated. Western blot analysis demonstrated that cortisone significantly increased the expression levels of SREBP-1 and its downstream proteins, including FAS and ACC [34]. However, co-incubation with Sap (or metyrapone as a positive control) reversed this effect. Specifically, compared with the cortisone stimulation group, the protein expression levels of SREPP-1, FAS, and ACC in the Sap group (6.0 μg/mL, incubation time: 24 h, cell viability > 90%) decreased by 65%, 80%, and 78%, respectively (Figure 4E–G). Additionally, the RT-PCR results indicated that cortisone treatment up-regulated the mRNA levels of SREBP-1, FAS, and ACC. Conversely, co-incubation with cortisone and Sap (or metyrapone as a positive control) led to a dose-dependent down-regulation of these mRNA levels. In the Sap group (6.0 μg/mL, incubation time: 24 h, cell viability > 90%), the mRNA levels of SREBP-1, FAS, and ACC were, respectively, 67%, 42%, and 100% lower than those in the cortisol-stimulating group (Figure 4H–J), which was consistent with the Western blot results and similar to the positive metyrapone group, indicating a desirable effect. The above results suggested that Sap inhibited lipid production in SZ95 sebocytes by modulating the 11β-HSD1/SREBP-1 pathways.

### 2.4. Sap Inhibits TNF-α-Stimulated Inflammation in NHEKs 

#### 2.4.1. Effects of Sap, TNF-α, and Metyrapone on NHEK Viability

**Figure 5:**
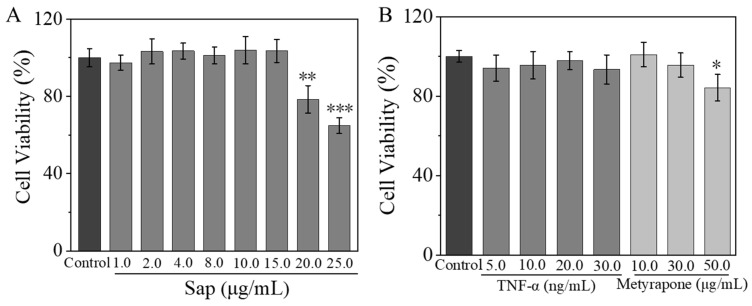
Effect of Sap, TNF-α, and metyrapone on cell viabilities of NHEKs. Toxicity effects of (**A**) Sap and (**B**) TNF-α and metyrapone. The error line represents the standard deviation of three independent culture results (*, ** and *** indicates *p* < 0.05, 0.01 and 0.001 vs. control group).

The toxicity concentration ranges of Sap, TNF-α, and metyrapone on NHEKs were also determined using the MTT assay. The cell viability remained above 90% for Sap concentrations ranging from 1.0 to 15.0 μg/mL, but dropped below 80% at concentrations of 20.0 and 25.0 μg/mL (Figure 5A). As depicted in Figure 5B, TNF-α maintained cell viability above 90% within the concentration ranges of 5.0 to 30.0 ng/mL. Metyrapone also kept cell viability above 90% at concentrations of 10.0 to 30.0 μg/mL, but it decreased below 80% at 50.0 μg/mL. Therefore, subsequent experiments were conducted using Sap concentrations of ≤ 15.0 μg/mL, TNF-α concentrations of ≤ 30.0 ng/mL, and metyrapone concentrations of ≤ 30.0 μg/mL.

#### 2.4.2. Sap Inhibits TNF-α-Stimulated Cortisol, IL-1β, IL-6, and IL-8 Production in NHEKs

Tumor necrosis factor-alpha (TNF-α) is a crucial cytokine that plays a significant role in regulating inflammatory responses and immune cell activity [35]. A study by Saori et al. [36] found that the stimulation of NHEK cells with TNF-α led to a significant increase in the expression of the 11β-HSD1 enzyme, as well as a notable rise in cortisol concentration in the cell culture supernatant. Based on these findings, NHEKs were stimulated with TNF-α to construct a stress model, and the cortisol concentration in the cell culture supernatant was measured using ELISA to determine the relationship between TNF-α concentration and cortisol secretion.

**Figure 6:**
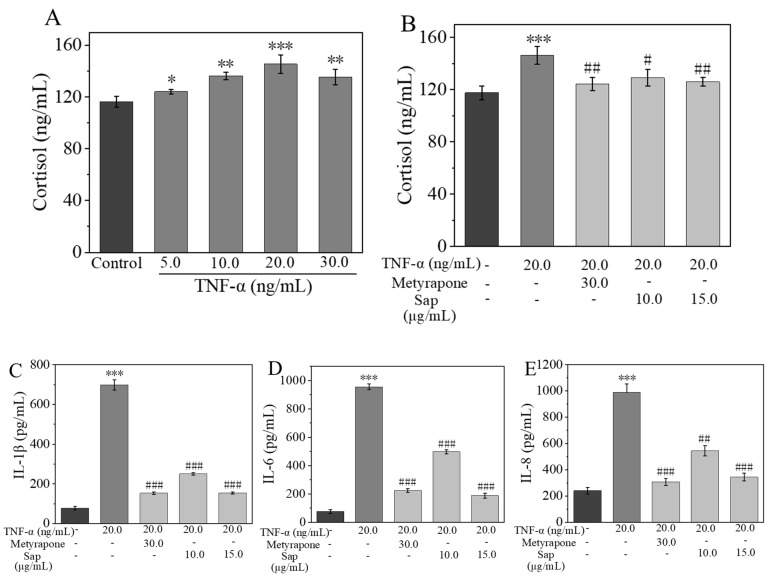
Sap inhibits TNF-α-stimulated cortisol (**A**,**B**), IL-1β (**C**), IL-6 (**D**), and IL-8 (**E**) production in NHEKs. The error line represents the standard deviation of three independent culture results (*, ** and *** indicate *p* < 0.05, 0.01, and 0.001 vs. control group; #, ##, and ### indicate *p* < 0.05, 0.01, and 0.001 vs. TNF-α-stimulated group).

It was observed that TNF-α enhanced cortisol secretion in a dose-dependent manner, with a 25% increase in secretion at a concentration of 20.0 ng/mL compared to the control group (Figure 6A). However, the co-culture of TNF-α with Sap significantly reduced cortisol secretion: in the Sap group (15.0 μg/mL, incubation time: 24 h, cell viability > 90%), cortisol secretion decreased by 13% compared to the TNF-α group, with an inhibitory effect similar to that of the positive control drug metyrapone (Figure 6B).

TNF-α-stimulated NHEKs are widely used as a cellular model for skin inflammation [37]. In this experiment, NHEKs were cultured for 24 h with TNF-α alone as well as a mixture of TNF-α and Sap or metyrapone, and then the concentrations of IL-1β, IL-6, and IL-8 in the cell supernatant were measured using ELISA. Compared to the control group, the TNF-α group exhibited significantly increased production of inflammatory factors of IL-1β, IL-6, and IL-8. Conversely, when co-culturing TNF-α with Sap (10.0 or 15.0 μg/mL, incubation time: 24 h, cell viability > 90%), the production of these three inflammatory factors were significantly inhibited, with an inhibitory effect comparable to that of the positive control of metyrapone (Figure 6C–E).

#### 2.4.3. Sap Inhibits Stress-Induced Inflammation via the 11β-HSD1/TLR2/NF-κB Pathways

**Figure 7:**
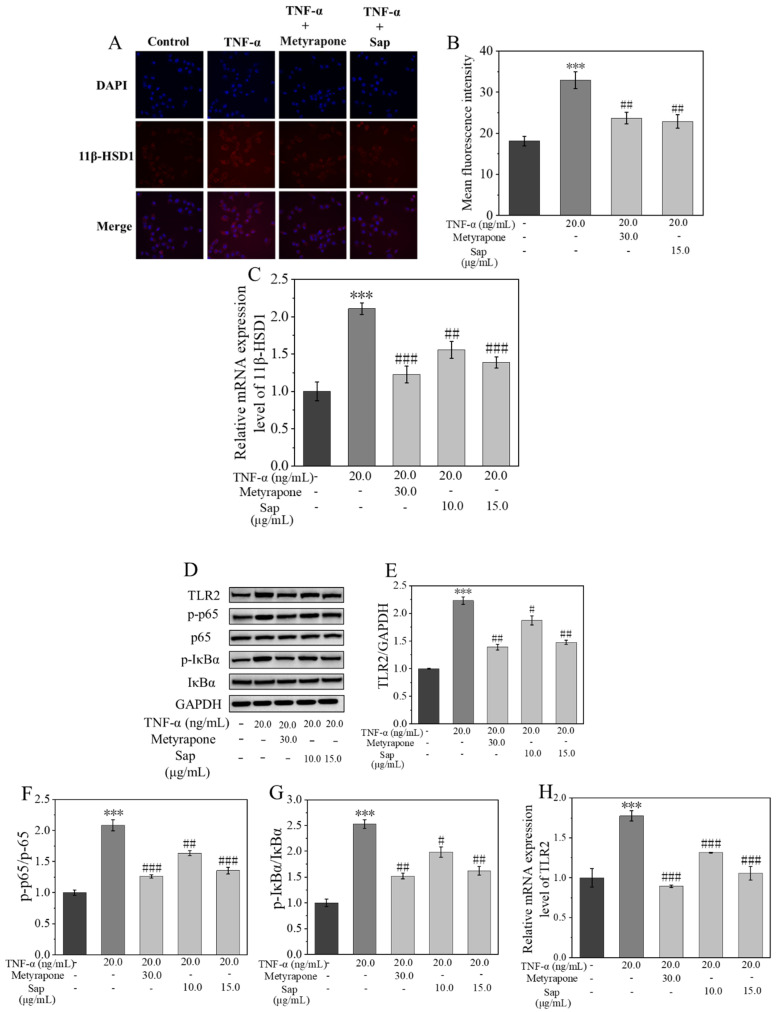
Sap inhibits TNF-α-stimulated inflammation via the 11β-HSD1/TLR2/NF-κB pathways. Immunofluorescence images (**A**,**B**) correspond to mean fluorescence intensity for 11β-HSD1 enzyme expression. (**C**) 11β-HSD1 mRNA expression levels; (**D**) Western blot of protein bands; (**E**–**G**) relative protein expression levels; (**H**) mRNA expression level of TLR2. The error line represents the standard deviation of three independent culture results (*** indicate *p* < 0.001 vs. control group; #, ##, and ### indicate *p* < 0.05, 0.01, and 0.001 vs. TNF-α-stimulated group).

11β-HSD1 plays a crucial role in the regulation of inflammatory factors. Its inhibition can effectively reduce inflammatory responses [36,38,39]. Therefore, the protein and mRNA expressions of the 11β-HSD1 enzyme were assessed using RT-PCR and immunofluorescence staining. As illustrated in Figure 7A–C, the protein and mRNA expression levels of the 11β-HSD1 enzyme were markedly elevated after cortisone treatment, a finding corroborated by the quantification of mean fluorescence intensity. Following co-culture of TNF-α with metyrapone or Sap (15.0 μg/mL, incubation time: 24 h, cell viability > 90%), both the protein and mRNA expression levels of the 11β-HSD1 enzyme were significantly reduced. These results indicated that Sap can effectively inhibit the TNF-α-stimulated increment in 11β-HSD1 enzyme activity, consequently diminishing cortisol secretion.

It is known that 11β-HSD1 is involved in the inflammatory process by modulating the expression of the upstream Toll-like receptor 2 (TLR2) signaling molecules [34]. Ultimately, the TLR2 receptor activates the NF-κB signaling pathway, which, in turn, initiates the transcription of inflammation-related genes [40,41]. Therefore, the Western blot technique was used to determine the expression of proteins related to the TLR2 and NF-κB pathways. As shown in Figure 7E,H, the protein and mRNA expression levels of TLR2 receptor significantly increased after TNF-α stimulation compared with the control group. The expression of TLR2 receptor decreased significantly after co-culture with metyrapone under TNF-α stimulation, suggesting that 11β-HSD1 was involved in the regulation of TLR2 receptor expression in NHEKs. Similarly, the expression of TLR2 receptor was also significantly decreased after the co-culture of TNF-α with Sap, suggesting that Sap regulated the expression of TLR2 receptor by inhibiting 11β-HSD1 enzyme activity [34]. In addition, we examined the activation of the NF-κB pathway after TNF-α stimulation. As shown in Figure 7F,G, phosphorylation levels of p65 and IκBα were significantly increased after TNF-α stimulation compared with the control group, indicating that the NF-κB pathway was activated. However, when Sap was added for co-culture, the activation of the NF-κB pathway was significantly inhibited, with inhibitory effects similar to those in the positive control with metyrapone group. This suggested that Sap exerted anti-inflammatory effects by inhibiting the 11β-HSD1/TLR2/NF-κB pathway.

## 3. Discussion

In modern society, people are facing increasing emotional pressure from life and work. Prolonged exposure to this state of stress activates the hypothalamic–pituitary–adrenal (HPA) axis, leading to excessive cortisol secretion, which, in addition to local stress, can disrupt skin homeostasis. Plant extracts have the merits of being safe, biocompatible, and easily available, and are attractive for improving skin problems caused by emotional and local stress. This study provides evidence that the Sap in tea seed meal inhibits stress-induced lipogenesis and inflammation in vitro.

Under stress, the body activates the hypothalamic–pituitary–adrenal (HPA) axis, which promotes the secretion of cortisol [3], and the physiologically active cortisol is converted from cortisone by the 11β-HSD1 enzyme, which can lead to lipid overproduction in the skin [12,20]. Based on this, we used cortisone to simulate the stress environment and to explore the effects of Sap on cortisol secretion and lipid production in SZ95 sebocytes. Cortisone was shown to promote cortisol secretion and lipid production in a concentration-dependent manner. Immunofluorescence staining and RT-PCR assays showed that cortisone significantly promoted the mRNA expression and protein levels of 11β-HSD1. In contrast, when Sap was co-cultured with cortisone, the expression of the 11β-HSD1 enzyme was significantly inhibited, and the efficiency of cortisol secretion and lipid synthesis were also decreased. In the Sap group (6.0 μg/mL, incubation time: 24 h, cell viability > 90%), the mRNA expression of 11β-HSD1 decreased by 47% compared with that of the cortisone group; the cortisol concentration in the culture supernatant decreased by 13.5 pg/mL, and the lipid synthesis rate decreased by 32%. This result was similar to that of the positive control, i.e., the drug metyrapone, suggesting that Sap reduced cortisol production and lipid synthesis by inhibiting the expression/activity of 11β-HSD1.

Sterol regulatory element-binding protein 1 (SREBP-1) is a key nuclear transcription factor that regulates the expression of downstream enzymes involved in lipid (such as cholesterol, fatty acid, and triglyceride) synthesis, including fatty acid synthase (FAS) and acetyl-CoA carboxylase (ACC) [42]. Previous studies have shown that in SZ95 sebocytes, 11β-HSD1 mediates the expression of SREBP-1, which, in turn, promotes lipogenesis [34]. Therefore, inhibiting the 11β-HSD1/SREBP-1 pathways can reduce lipid production within the cells. The Western blot results showed that cortisone significantly promoted the protein expression of SREBP-1 and its downstream targets of FAS and ACC. However, when Sap was added to co-culture with cortisone, this promoting effect was significantly inhibited. In the Sap group (6.0 μg/mL, incubation time: 24 h, cell viability > 90%), the protein expression of SREBP-1 and its downstream targets of FAS and ACC decreased by 65%, 80%, and 78%, respectively, compared with the cortisone group, which was similar to the effect of the positive control of metyrapone. In addition, the RT-PCR results showed the same trend as the Western blot results. In the Sap group (6.0 μg/mL, incubation time: 24 h, cell viability > 90%), the mRNA expression of SREBP-1 and its downstream targets of FAS and ACC decreased by 67%, 42%, and 100%, respectively, compared with the cortisone stimulative group. This suggested that TSS regulated lipid production in SZ95 sebocytes through the 11β-HSD1/SREBP-1 pathway. We previously showed that this saponin reduced sebum synthesis via the AMPK/mTOR axis [24]; here, we further demonstrated that it exerted additional lipid-lowering effects through the 11β-HSD1/SREBP-1 pathway. All of these results indicate that this kind of saponin might act as a multi-target ingredient for inhibiting lipid synthesis through versatile pathways.

TNF-α is a pleiotropic cytokine mainly produced by macrophages, monocytes, and T cells, which can promote the release of inflammatory factors and is often used to construct cellular inflammation models [37,43]. In order to simulate the stress-induced inflammatory effect of the skin, TNF-α was used to stimulate NHEKs. The results showed that TNF-α could significantly increase the secretion of cortisol. In addition, the concentrations of the inflammatory cytokines IL-1β, IL-6, and IL-8 in the cell supernatant were detected by ELISA, and it was found that Sap (10.0 or 15.0 μg/mL, incubation time: 24 h, cell viability > 90%) could significantly inhibit the production of TNF-α-stimulated inflammatory factors, and this inhibitory effect was comparable to that of the positive control of metyrapone.

Through RT-PCR and immunofluorescence staining analysis, we found that upon TNF-α stimulation, the mRNA and protein expression levels of 11β-HSD1 were significantly up-regulated, and the secretion of cortisol was also significantly increased. However, this phenomenon was significantly reversed when Sap was co-cultured with TNF-α. Specifically, in the Sap group (15.0 μg/mL, incubation time: 24 h, cell viability > 90%), the mRNA expression of 11β-HSD1 was reduced by 72% and cortisol secretion was decreased by 13% compared to that of the group treated with TNF-α alone, with inhibitory effects comparable to those of the positive control. These results suggest that the 11β-HSD1 enzyme played an important role in the regulation of inflammation.

TLR2 is an important member of the pattern recognition receptor (PRR) family capable of recognizing multiple pathogen-associated molecular patterns (PAMPs) and damage-associated molecular patterns (DAMPs), and plays a key role in innate immune and inflammatory responses. When TLR2 recognizes its ligand, MyD88 is activated, which leads to the degradation of IκB by activating the IKK complex, releasing NF-κB dimers (e.g., p65/p50) into the nucleus and activating the NF-κB signaling pathway [40,41]. Based on the findings of our previous studies [34], we further investigated the influence of Sap on the expression of TLR2 and subsequent effect on the NF-κB pathway. The Western blot and RT-PCR results showed that TNF-α significantly promoted the protein and mRNA expression of TLR2 and activated the NF-κB pathway. However, this phenomenon was significantly reversed when Sap was co-cultured with TNF-α. Specifically, compared with the TNF-α-alone group, the protein and mRNA expressions of TLR2 in the Sap group (15.0 μg/mL, incubation time: 24 h, cell viability > 90%) decreased by 76% and 72%, respectively, while the p-p65/p65 and p-IκBα/IκBα ratios were reduced by 73% and 91%, respectively. These inhibitory effects were comparable to those of the positive control of metyrapone. These results suggest that Sap effectively inhibited TNF-α-stimulated inflammation by inhibiting the 11β-HSD1/TLR2/NF-κB signaling pathway.

**Figure 8:**
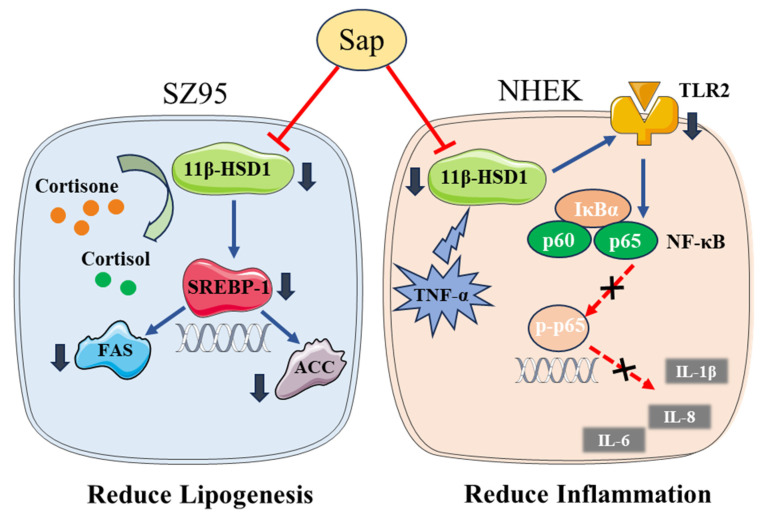
In SZ95 sebocytes, Sap inhibits cortisol and sebum production by regulating the 11β-HSD1/SREBP-1 pathway. In NHEKs cells, Sap inhibits the production of inflammatory factors by regulating the 11β-HSD1/TLR2/NF-κB pathway.

The inhibitory effect of Sap on the excessive production of sebum and inflammatory factors may be the combined effect of saponins and polyphenols in Sap [44,45,46,47]. However, future work is still needed to reveal the contribution of each constituent to clarify which molecule chiefly underlies Sap’s efficacy, validate the findings through clinical trials, and explore whether additional mechanisms remain to be identified. Figure 8 illustrates the mechanism of action of Sap in SZ95 and NHEK cells.

## 4. Materials and Methods

### 4.1. Extraction and Purification of Sap from Tea Seed Meal

#### 4.1.1. Extraction of Sap

Tea (*Camellia sinensis*) seed meal was obtained from a tea (*Camellia sinensis*) tree planted by a tea Hunan Anhua Dark Tea Group Co., Ltd. r in Anhua, Hunan, China, a city located at a latitude of 28 degrees north. The tea is very famous in China and the region is known as the “Town of Dark Tea”. Tea (*Camellia sinensis*) seed meal was dissolved in 70% ethanol (Sinopharm Group Chemical Reagent Co., Ltd., Shanghai, China) at a solid-to-liquid ratio of 1:5 (*w*/*v*) and stirred at 60 °C for 4 h. The resulting mixture was filtered through a 0.22 μm membrane (Sinopharm Group Chemical Reagent Co., Ltd., Shanghai, China) to collect the filtrate, which was then concentrated to form a viscous solution. Then, the solution was washed three times with petroleum ether (Sinopharm Group Chemical Reagent Co., Ltd., Shanghai, China) to remove oil-soluble impurities. Subsequently, n-butanol (Sinopharm Group Chemical Reagent Co., Ltd., Shanghai, China) was added to the liquid phase for Sap extraction until the extract phase appeared clear. Finally, the extracts were collected by centrifugation and concentrated under reduced pressure to yield a viscous solution.

#### 4.1.2. Isolation and Purification of Sap

The crude Sap solution was purified using macroporous resin column chromatography following the method of Yu et al. [27]. Briefly, the viscous solution was diluted with deionized water and loaded onto a D101 macroporous resin (Sinopharm Group Chemical Reagent Co., Ltd., Shanghai, China) at a mass ratio of approximately 1:10. The column was sequentially eluted with deionized water, 0.2% NaOH solution (Sinopharm Group Chemical Reagent Co., Ltd., Shanghai and China), and 20% ethanol (Sinopharm Group Chemical Reagent Co., Ltd., Shanghai and China). Finally, the target fraction was collected by elution with 80% ethanol (Sinopharm Group Chemical Reagent Co., Ltd., Shanghai and China), concentrated and freeze-dried to obtain purified Sap powder.

#### 4.1.3. Total Saponin Content Determination

The vanillin–concentrated sulfuric acid method was employed for the quantitative determination of saponin content, following the procedure described in reference [48] with some modifications. Briefly, 20 μL of oleanolic acid solution (Aladdin Industrial Corporation Shanghai, China) at gradient concentrations was accurately pipetted into a 10 mL EP tube and evaporated to dryness. Then, 0.3 mL of 5% vanillin (Aladdin Industrial Corporation, Shanghai, China) in glacial acetic acid (Aladdin Industrial Corporation, Shanghai, China) and 0.7 mL of concentrated sulfuric acid (Aladdin Industrial Corporation, Shanghai, China) were added to the dried residue. The mixture was heated in a 70 °C water bath for 15 min. After the reaction, the mixture was immediately cooled to room temperature in an ice water bath. Finally, 5 mL of glacial acetic acid was added to dilute the sample. A blank was prepared using the same reaction system without saponin. The absorbance at 540 nm was measured using an ultraviolet spectrophotometer (Thermo Fisher Scientific, Waltham, MA, USA), and a standard curve was constructed. The same procedure was applied to determine the saponin content in the sample (Sap).

#### 4.1.4. Determination of Total Phenol Content

The polyphenol content was determined using the Folin–Ciocalteu assay according to the method described in reference [49] with some modifications. Briefly, 1 mL of gallic acid solution (Aladdin Industrial Corporation, Shanghai, China) at gradient concentrations was accurately pipetted into a 10 mL EP tube, followed by the addition of 4 mL of 10% Folin–Ciocalteu reagent (Aladdin Industrial Corporation, Shanghai, China). The mixture was allowed to react at room temperature for 5 min. Then, 4 mL of 7.5% sodium carbonate (Na_2_CO_3_) solution (Sinopharm Group Chemical Reagent Co., Ltd., Shanghai, China) was added, and the reaction proceeded at room temperature for 60 min. A blank was prepared using the same reaction system without gallic acid. The absorbance at 765 nm was measured using a UV spectrophotometer, and a standard curve was constructed. The same procedure was applied to determine the polyphenol content in the sample (Sap).

#### 4.1.5. Structure Identification of Sap from Tea Seed Meal

A 10 mg sample of Sap was dissolved in 20 mL of 80% ethanol, and its UV-vis absorption spectra were analyzed using a Genesys 10S UV-VIS spectrophotometer (scanned between 200 and 400 nm; Thermo Fisher, Waltham, MA, USA). For FT-IR analysis, Sap powder was homogenized with KBr crystals, pressed into pellets, and examined using a Nicolet iS50 FTIR spectrometer (Thermo Fisher, Waltham, MA, USA). To further elucidate the structure, Sap was subjected to ultra-high-performance liquid chromatography (UHPLC) coupled with an X500R high-resolution mass spectrometer (AB SCIEX, Framingham, MA, USA), with data acquisition using OS software (Version 2.2.0.5738, SCIEX, Foster City, CA, USA).

Chromatographic conditions: The separation was carried out on a Waters ACQUITY UPLC^®^ BEH C18 column (1.7 μm, 2.1 × 100 mm) at 50 °C, with an injection volume of 4.0 μL. The mobile phase comprised (A) 0.1% aqueous formic acid and (B) 0.1% formic acid in acetonitrile, delivered at a constant flow rate of 0.3 mL/min. The following gradient program was employed: initial 5% B (0 to 2 min), linear increase to 99% B (2 to 10 min), isocratic hold at 100% B (10 to 20 min), and re-equilibration to initial conditions at 5% B (20 to 23 min).

Mass spectrometry parameters: The ion source temperature was set at 550 °C with a gas curtain flow rate of 35 psi. In the MS scan, the declustering potential (DP) was 80 V, and the collision energy (CE) was 10 eV. For MS/MS mode, the collision energy (CE) was 40 ± 15 eV. The mass scanning ranges were 70–1500 Da for primary MS and 50–1500 Da for secondary MS.

### 4.2. Cell Culture

The human immortalized sebaceous cell line SZ95 (Provided by Professor Christos C Zouboulis, Germany) [50] and NHEKs (Shenzhen Haodi Huatuo Biotechnology Co., Ltd., Shengzhen, China) were cultured in DMEM supplemented with 10% fetal bovine serum (Gibco, Carlsbad, CA, USA), 1.0 × 105 U/L penicillin (Shanghai Biyun Tian Biotechnology Co., Ltd., Shanghai, China), and 100 mg/L streptomycin (Shanghai Biyun Tian Biotechnology Co., Ltd., Shanghai, China). Cultures were maintained at 37 °C with 5% CO_2_ under saturated humidity. For subculture, cells were treated with trypsin–EDTA solution containing 0.25% EDTA (Gibco) (Shanghai Youningwei Biological Technology Co., Ltd., Shanghai, China) and passaged in fresh complete medium as described above.

### 4.3. MTT Assay for Cell Viability

To evaluate the cytotoxic effects of Sap (Made by oneself, city and country), cortisone (Aladdin Industrial Corporation, Shanghai, China), and TNF-α (Aladdin Industrial Corporation, Shanghai, China) using an MTT assay, we prepared serial dilutions from the following stock solutions: (1) Sap (1.0 mg/mL in DMEM (Shanghai Youningwei Biological Technology Co., Ltd., Shanghai, China), prepared by dissolving 10 mg in 10 mL); (2) cortisone (50 mg/mL in DMSO, prepared by dissolving 100 mg in 2.0 mL); and (3) TNF-α (1.0 μg/mL in PBS (Shanghai Youningwei Biological Technology Co., Ltd., Shanghai, China), prepared by dissolving 10 μg in 10 mL). Each substance was then diluted to achieve the desired test concentrations.

SZ95 sebocytes and NHEKs in logarithmic growth phase were trypsinized, counted, and seeded into 96-well plates at a density of 1 × 10^5^ cells/mL per well (100 μL/well). Following 24 h of incubation at 37 °C in a 5% CO_2_ incubator (Shanghai Bosun Medical Biological Instrument Co., Ltd., Shanghai, China), the experimental groups were treated with 100 μL of sample solutions of varying mass concentrations dissolved in DMEM (prepared as described in Section 4.3), while the blank group received 100 μL of DMEM. Following an additional 24 h of culture, the supernatant was gently decanted. After 24 h of treatment, the culture supernatants were carefully aspirated and replaced with 3-(4,5-dimethylthiaxolone-2-yl)-2,5-diphenyl tetra-zoliumbromide (MTT) (Sigma-Aldrich, Saint Louis, MO, USA) (100 μL, 0.5 mg/mL) and incubated for another 4 h. After the above incubation medium was gently removed, 100 μL of dimethyl sulfoxide (DMSO; Sinopharm, Beijing, China) was added to dissolve the formazan crystals. The absorbance at 490 nm was measured with a microplate reader (Tecan Inﬁnite 200Pro; Maennedorf, Switzerland). Cell viability was calculated using the following formula:

Cell viability (%) = (OD_T_/OD_B_) × 100% (1)

where OD_T_ and OD_B_ represent the average OD values of the experimental and the blank groups at 490 nm, respectively.

### 4.4. Effects of Sap on Cortisone-Induced Cortisol Conversion and Lipid Secretion in SZ95

To examine cortisone’s impact on cortisol production, SZ95 sebocytes in the logarithmic growth phase were seeded (at a density of 1 × 10^5^ cells/mL with a volume of 100 μL/well) into a black-bordered 96-well plate and cultured for 24 h at 37 °C in a 5% CO_2_ incubator. The control group was treated with DMEM and the experimental groups were treated with different concentrations (5.0, 10.0, and 20.0 μg/mL) of cortisone in DMEM. After 24 h of incubation, the cell supernatant was collected for measuring cortisol levels using an ELISA kit (Shanghai Youningwei Biological Technology Co., Ltd., Shanghai, China).

Following supernatant removal, the cells were stained with 100 μL of Nile Red staining solution (Shanghai Youningwei Biological Technology Co., Ltd., Shanghai, China) (10 μg/mL) and incubated at 37 °C in the dark for 15 min. Lipid droplets were visualized using a fluorescence microscope (Chongqing Optec Instrument Co., Ltd., Chongqing, China) and corresponding fluorescence intensities for quantitative analysis were measured using a multifunctional plate reader (BioTek Instruments, Inc, Winooski, VT, USA) under the excitation/emission wavelengths of 485 and 565 nm, respectively. The lipid synthesis ratio of SZ95 sebocytes was calculated by referring to the following formula:

Lipid synthesis ratio (%) = (FI_T_/FI_B_) × 100% (2)

where FI_T_ and FI_B_ are the average fluorescence intensities of the experimental and control groups, respectively.

To evaluate the modulatory effects of Sap on the cortisone-induced cortisol conversion and sebum secretion in SZ95 cells, the cells were divided into three treatment groups: the control group received 100 μL of DMEM, the cortisone group received 100 μL of a DMEM solution containing 20.0 μg/mL cortisone, and the experimental group received 100 μL mixtures of cortisone (20.0 μg/mL) and metyrapone (30.0 μg/mL) or Sap (4.0 or 6.0 μg/mL). 

### 4.5. Effects of Sap on the Secretion of Cortisol and Inflammatory Factors in NHEKs Induced by TNF-α

NHEK seeding was performed as described in Section 4.3. After incubating cells with varying TNF-α concentrations (5.0, 10.0, 20.0, and 30.0 ng/mL) for 24 h, the cortisol and inflammatory cytokine (IL-1β, IL-6, and IL-8) concentrations in the culture supernatant were collected and measured using ELISA kits. These measurements enabled the evaluation of the dose-dependent effects of TNF-α on cortisol or inflammatory cytokine secretion levels. 

Next, the effect of Sap on TNF-α-induced cortisol conversion in NHEK cells was also examined. The control group received 100 μL of DMEM; the TNF-α group received 100 μL of DMEM containing 20.0 ng/mL TNF-α; and the experimental groups received 100 μL of mixtures of TNF-α (20.0 ng/mL) with either metyrapone (30.0 μg/mL) or Sap (10.0 or 15.0 μg/mL). 

### 4.6. Immunofluorescence

To evaluate the regulatory effects of Sap on 11β-HSD1 expression, immunofluorescence staining was performed with SZ95 sebocytes and NHEKs. Specifically, the SZ95 or NHEK cells were seeded at 7 × 10^4^ cells/mL in confocal dishes and cultured for 24 h, followed by the treatment with cortisone alone, the mixture of cortisone and Sap, or the mixture of cortisone and metyrapone for SZ95 sebocytes; for TNF-α alone, the mixture of TNF-α and Sap or the mixture of TNF-α and metyrapone were used for NHEKs. After 24 h of treatment, the cells were washed with PBS and fixed for 15 min. Then, the dishes were blocked by immunostaining blocking solution (Shanghai Youningwei Biological Technology Co., Ltd., Shanghai, China) for 1 h at room temperature before overnight incubation at 4 °C with the 11β-HSD1 primary antibody. This procedure was followed by a two-hour incubation with the Alexa fluor 594 labeled secondary antibody. Nuclei were stained with DAPI-containing mounting medium for 10 min and imaged using a laser confocal microscope, while mean fluorescence intensity was measured with ImageJ (ImageJ 1.53k, Wayne Rasband, Bethesda, MD, USA).

### 4.7. RT-PCR Analysis of mRNA Expression of Related Genes

SZ95 and NHEK cells (1.5 × 10⁵ cells /mL, 6 mL per bottle) were inoculated into T25 culture bottles and allowed to adhere for 24 h. Then, SZ95 sebocytes were incubated with cortisone (20.0 μg/mL) alone or a mixture of cortisone (20.0 μg/mL) and Sap (4.0 or 6.0 μg/mL) or metyrapone (30.0 μg/mL); NHEKs were incubated with TNF-α (20.0 ng/mL) or a mixture of TNF-α (20.0 ng/mL) and Sap (10.0 or 15.0 μg/mL) or metyrapone (30.0 μg/mL), respectively. After an incubation for 24 h, the cells were harvested using a trypsin solution containing 0.25% EDTA, followed by termination of digestion with DMEM. Cell suspensions were centrifuged at 1200 rpm/min for 5 min to collect cell precipitates. The total RNA was extracted using an RNeasy RNA extraction kit (Shanghai Youningwei Biological Technology Co., Ltd., Shanghai, China) and reverse-transcribed into cDNA for subsequent experiments. Subsequently, PCR amplification was performed using the diluted cDNA (approximately 200 ng/µL) as the template. The PCR amplification program was as follows: initial denaturation at 94 °C for 30 s, followed by 94 °C for 5 s and 60 °C for 30 s for 40 cycles; melted at 95 °C for 15 s and 60 °C for 1 min, and then the temperature was increased at rate of 0.3 °C every 15 s, ending at 95 °C for 15 s. Finally, the relative expression was normalized using the 2^−ΔΔCt^ method. In SZ95 cells, GAPDH was used as the internal reference gene, and in NHEKs, β-actin was used as the internal reference gene. The primers used are shown in Table 2 and Table 3.

### 4.8. Western Blotting

Cell culture and grouping followed the protocol described in Section 4.7. After 24 h of treatment, cell precipitation was collected by centrifugation, and total protein was extracted using RIPA lysis, with the protein quantified by a BCA protein assay kit (Shanghai Youningwei Biological Technology Co., Ltd., Shanghai, China). The obtained protein samples (30 μg each) were loaded and separated on SDS-PAGE gels (Shanghai Youningwei Biological Technology Co., Ltd., Shanghai, China) and then transferred onto PVDF membrane. After blocking with 5% skim milk (Shanghai Biyun Tian Biotechnology Co., Ltd., Shanghai, China) or 1% bovine serum albumin (Beyotime, Shanghai, China), the membranes were incubated with the primary antibodies (against 11β-HSD1, SREBP-1, FAS, ACC, TLR2, p65, p-p65, IκBα, or p-IκBα antibodies at a dilution ratio of 1:1000) at 4 °C overnight. The next day, following the wash with TBST (Shanghai Biyun Tian Biotechnology Co., Ltd., Shanghai, China), the membranes were incubated with the HRP-labeled secondary antibodies (at a dilution ratio of 1:3000) at room temperature for 1 h and protein bands were detected using an ECL kit (Shanghai Biyun Tian Biotechnology Co., Ltd., Shanghai, China) (Beyotime) and imaged with a Chemi DOC XRS+ gel imaging system (Shanghai Biyun Tian Biotechnology Co., Ltd., Shanghai, China). Protein expression levels were normalized to GAPDH (for SZ95 cells) or β-actin for NHEKs. All of the antibodies used in the experiment were sourced from the Wuhan Sanying Biotechnology Co., Ltd. Wuhan Sanying, Wuhan, Hubei, China. 

### 4.9. Statistical Analysis

The data analysis was carried out by GraphPad (GraphPad Prism 10.3.1, GraphPad Software, LLC, Boston, MA, USA) and data were expressed in the form of mean ± standard deviation. The comparison between groups was assessed through an analysis of variance (ANOVA) followed by the Tukey test. A *p*-value less than 0.05 was considered to be statistically significant.

## 5. Conclusions

Saponin from tea (*Camellia sinensis*) seed meal is a natural product with under-developed cosmetic applications. This work validated the unique emotional and local stress-relieving characteristics on skin and the mechanism of Sap from tea (Anhua, Hunan Province, China) seed meal in terms of its sebum control and anti-inflammatory effects. It was found that Sap in the concentration range of 4.0–15.0 μg/mL could inhibit the conversion of cortisol in SZ95 and NHEK cells under stress, mainly by inhibiting the expression of the 11β-HSD1 enzyme. Furthermore, Sap could also reverse the elevation in lipid levels induced by cortisone in SZ95 sebocytes, mainly through the SREBP-1 signaling pathway. Moreover, Sap exerts anti-inflammatory effects on TNF-α-stimulated NHEKs in the secretion of inflammatory cytokines, such as IL-1β, IL-6, and IL-8, by inhibiting the 11β-HSD1 enzyme involved TLR2/NF-κB signaling pathway. These findings suggest that Sap from tea seed meal has a desirable effect for alleviating emotional and local stress-invoked cutaneous problems, which provides an extended application of Sap from tea seed meal in cosmetics, especially in mitigating detrimental effects caused by stress. However, the commercialization of Sap still requires further research, including in vivo efficacy validation, safety evaluation, and formulation stability testing.

## Figures and Tables

**Table 1:** UHPLC-MS analysis for the structure of Sap from tea seed meal.

Compound	Rt/(min)	Molecular Formula	Structural Formula
1	5.57	C_47_H_74_O_17_	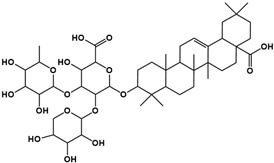
2	6.72	C_43_H_68_O_14_	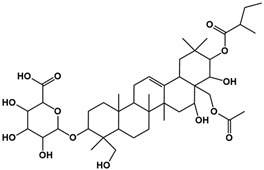
3	8.56	C_42_H_68_O_14_	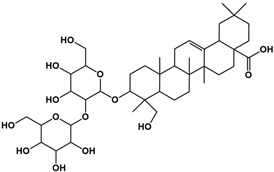
4	10.29	C_48_H_78_O_19_	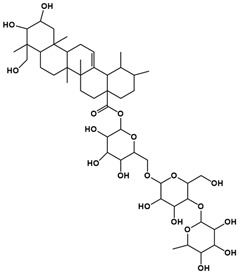
5	12.36	C_41_H_66_O_13_	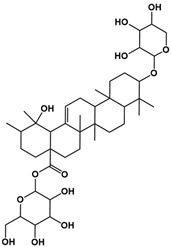
6	15.87	C_42_H_70_O_12_	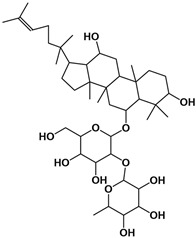

**Table 2:** Primer design for SZ95 cells.

Primer Name	Primer Sequence
11β-HSD1	F: 5′-TGGAGCAGCCTCAGCACACTAC-3′
	R: 5′-GTGTTGGTGATGTGGTTGAGAATG-3′
SREBP-1	F: 5′-CCCACTTCATCAAGGCAGACTCG-3′
	R: 5′-CACTCACCAGGGTCGGCAAAG-3′
FAS	F: 5′-TGGCACACATCCTGGGCATC-3′
	R: 5′-CAGACCATCCTCCTTGGGCGT-3′
ACC	F: 5′-ACAACGCAGGCATCAGAAGATTATT-3′
	R: 5′-CCACCATTTTGGCAAGTTTCACC-3′
GAPDH	F: 5’-GAAGGTGAAGGTCGGAGTC-3’
	R: 5’-GAAGATGGTGATGGGATTTC-3’

**Table 3:** Primer design for NHEKs.

Primer Name	Primer Sequence
11β-HSD1	F: 5′-TGGAGCAGCCTCAGCACACTAC-3′
	R: 5′-GTGTTGGTGATGTGGTTGAGAATG-3′
TLR2	F: 5′-CTTCTCCCATTTCCGTCTTTTTG-3′
	R: 5′-TCTTGGTGTTCATTATCTTCCGC-3′
β-actin	F: 5′-TGGCACCCAGCACAATGAA-3′
	R: 5′-GAAGCATTTGCGGTGGACG-3′

## Data Availability

The data used to support the findings of this study are available from the corresponding author upon request.

## Supplementary Materials

The following supporting information can be downloaded at https://www.mdpi.com/article/10.3390/molecules30193844/s1: Figure S1. The secondary mass spectrum of compound **1**; Figure S2. The secondary mass spectrum of compound **2**; Figure S3. The secondary mass spectrum of compound **3**; Figure S4. The secondary mass spectrum of compound **4**; Figure S5. The secondary mass spectrum of compound **5**; Figure S6. The secondary mass spectrum of compound **6**.

## Abbreviations

Sap	Saponin
SZ95	Human sebaceous gland cell line SZ95
NHEKs	Normal human epidermal keratinocytes
