# Supplementary material for: Saponin from Tea (Camellia sinensis) Seed Meal Attenuates Cortisol-Induced Lipogenesis and Inflammation in Human Cells
Source: Molecules. 2025 Sep 23;30(19):3844. doi: 10.3390/molecules30193844 (PMC12525551; doi:10.3390/molecules30193844)
Supplement: Supplementary file 1 [file molecules-30-03844-s001.zip › molecules-3833642-supplementary.pdf]

Supporting Information for

Saponin from Tea (*Camellia sinensis*) Seed Meal Attenuates Cortisol-Induced Lipogenesis and Inflammation in Human Cells

Jian Li [1], Lu-Yao Zhang [1], Yuan-Cheng Huang [2], Jian-Ming Deng [2], Min Yu [2], Christos C Zouboulis [3], Jin-Hua Li [1], Guang-Li Wang [1,*] and Jing Wang [1,*]

[1] School of Chemical and Material Engineering, Jiangnan University, Wuxi 214122, China
[2] Guangzhou Huashi Cosmetic Technology CO., LTD, Guangzhou, Guangdong 510000, China
[3] Department of Dermatology, Venereology, Allergology and Immunology, Staedtisches Klinikum Dessau, Brandenburg Medical School Theodor Fontane and Faculty of Health Sciences Brandenburg, Dessau D-06847, Germany
* Correspondence: glwang@jiangnan.edu.cn (G.-L.W.); jingwang@jiangnan.edu.cn (J.W.)

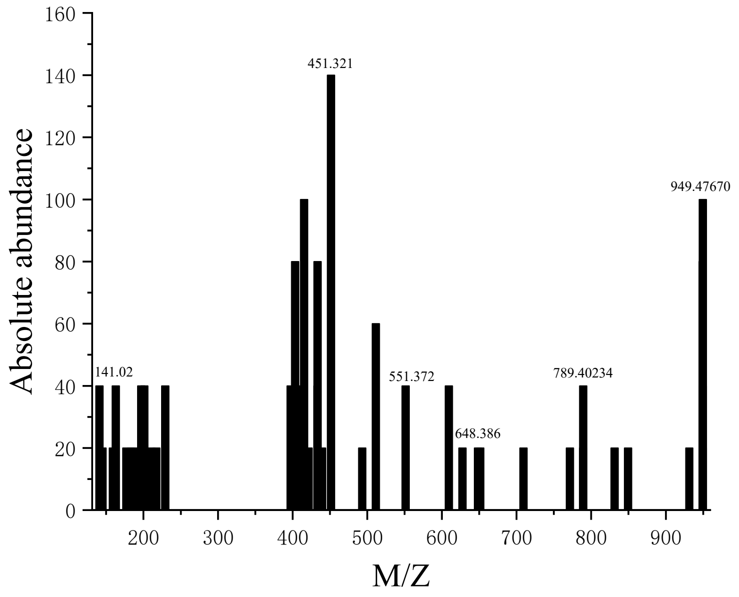

Figure S1. The secondary mass spectrum of compound 1.

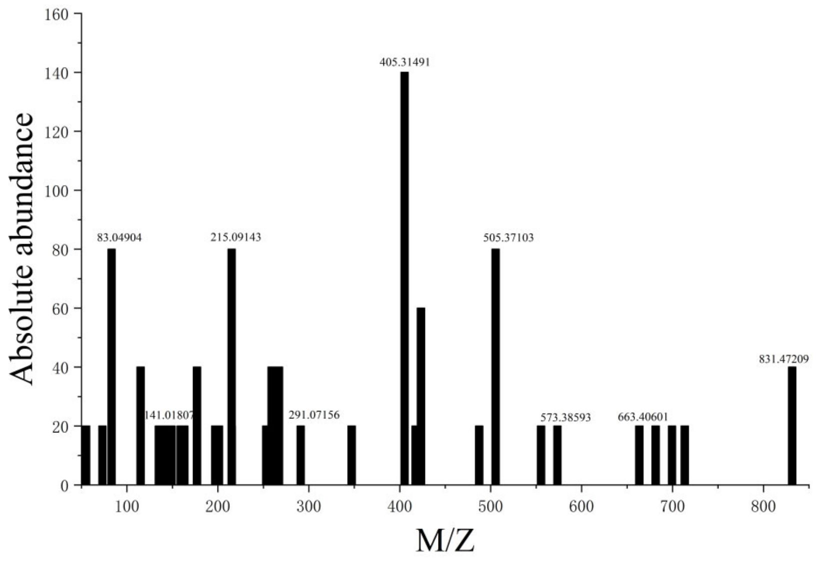

Figure S2. The secondary mass spectrum of compound 2.

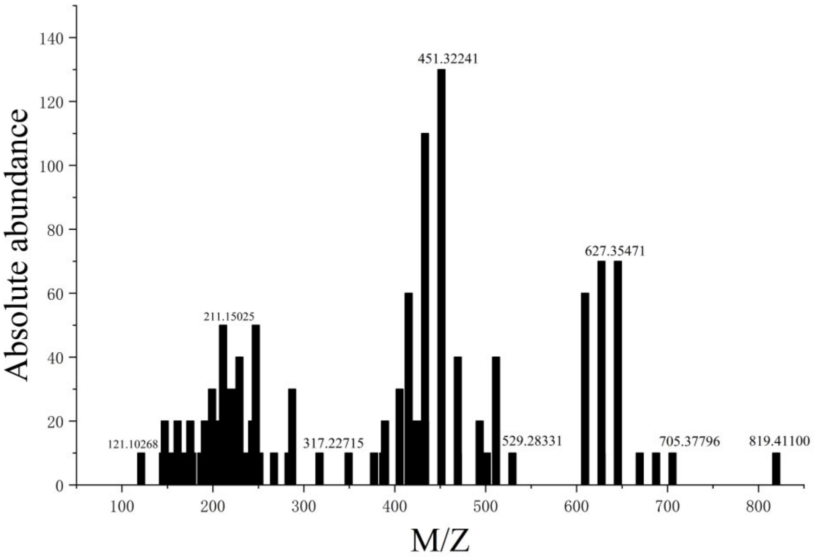

Figure S3. The secondary mass spectrum of compound 3.

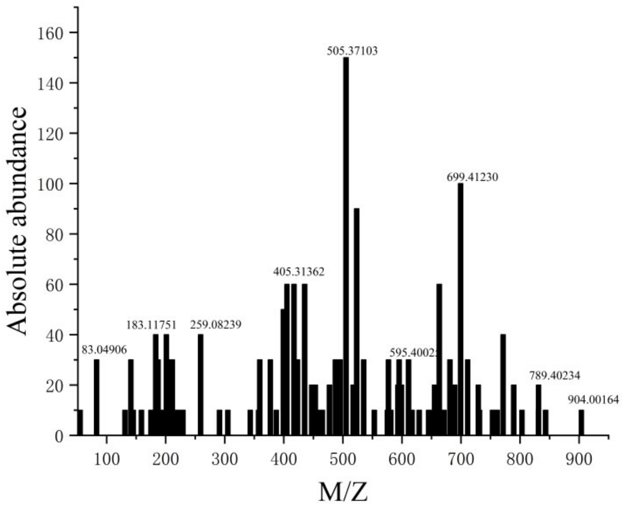

Figure S4. The secondary mass spectrum of compound 4.

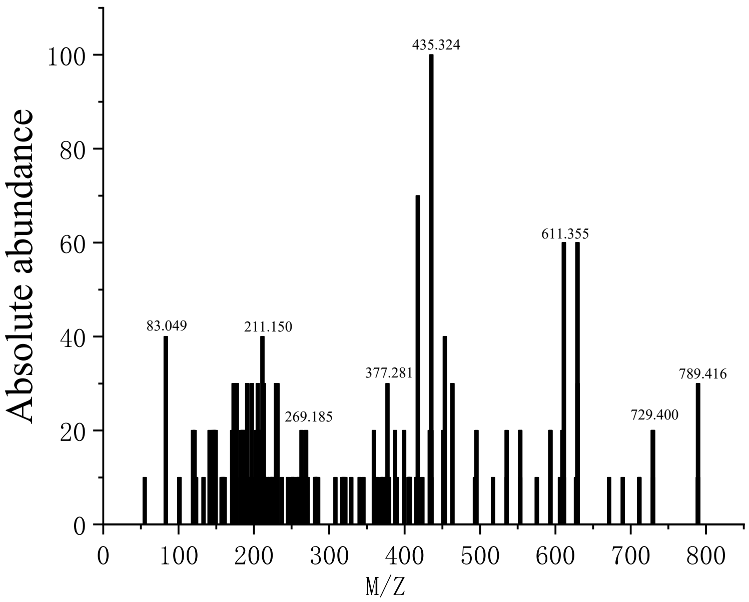

Figure S5. The secondary mass spectrum of compound 5.

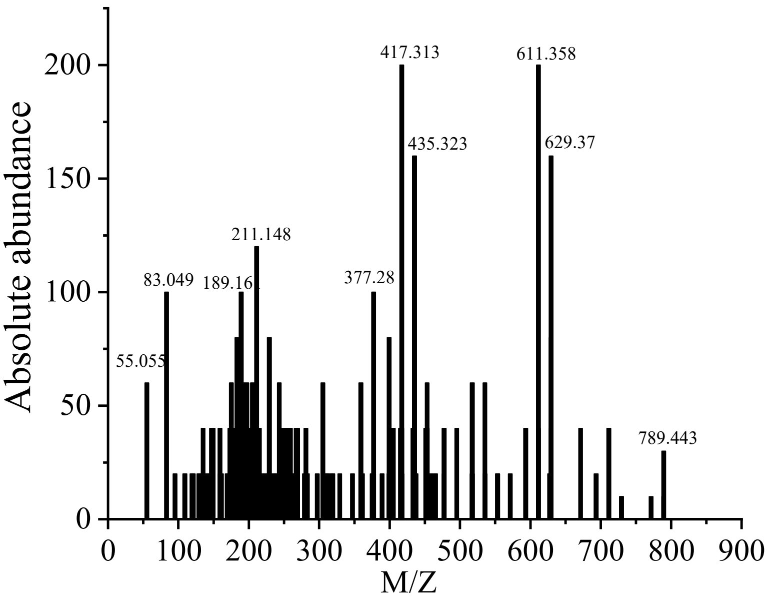

Figure S6. The secondary mass spectrum of compound 6.

Compounds 2, 3, 5 and 6 all afford fragment ions near m/z 211, characteristic of the Δ^{12}-oleanene skeleton ($C_{14}H_{19}O_2$). Compounds 1 and 3 additionally share the m/z 451 fragment, corresponding to [aglycone-COOH]$^-$, whereas compounds 2 and 4 exhibit m/z 405.31, generated by a further loss of HCOOH from [aglycone-COOH]$^-$, reflecting a McLafferty-type rearrangement that expels formic acid from the C-28 carboxyl group. For compound 6, whose precursor ion is m/z 627, the MS^2 spectrum

shows m/z 417, arising from the combined loss of the entire sugar chain (Glc + GlcA) as a 210-Da unit via α-cleavage of the C-28 ester, while the peak at m/z 611 results from the elimination of a single oxygen atom from the 23-hydroxyl group, yielding a 23-keto-aglycone fragment.
